# Can agricultural digitalization improve agricultural export quality?

**DOI:** 10.1371/journal.pone.0347210

**Published:** 2026-05-12

**Authors:** Zhichao Jiang, Xiaorun Wang, Pengling Liu

**Affiliations:** 1 School of Economics and Management, Anhui Agricultural University, Hefei, China; 2 School of Rural Revitalization, Anhui Agricultural University, Hefei, China; Guilin University of Aerospace Technology, CHINA

## Abstract

In the context of an evolving international trade landscape, agricultural digitalization represents a critical pathway for enhancing agricultural export quality (AEQ) and accelerating the development of a modern agricultural powerhouse. Using panel data from 34 provincial administrative regions in China from 2001 to 2024, this study constructs a comprehensive evaluation index system for agricultural digitalization and conducts a multidimensional analysis employing an individual, time two-way fixed effects model, a mediation effects model, and a moderation effects model. The results indicate that: (1) agricultural digitalization significantly promotes improvements in AEQ, and this conclusion remains robust after addressing endogeneity concerns and conducting a series of robustness tests; (2) accelerating agricultural technological progress, improving the level of agricultural human capital, and enhancing agricultural logistics efficiency constitute important transmission pathways through which agricultural digitalization affects AEQ; (3) the effect of agricultural digitalization on AEQ exhibits multidimensional heterogeneity, with stronger marginal effects observed in eastern regions, major grain-producing areas, and regions with higher levels of marketization; and (4) policy support and market openness play significant moderating roles in the relationship between agricultural digitalization and AEQ. Based on these findings, this study proposes several policy recommendations, including accelerating the development of agricultural digitalization, promoting agricultural technological progress, and establishing a scientific and regionally tailored agricultural digital system that reflects local conditions. These recommendations provide a scientific basis for policymaking aimed at leveraging agricultural digitalization to enhance AEQ.

## 1. Introduction and references

The intensification of global demand for high-quality agricultural development, together with profound changes in international trade patterns, has made agricultural exports an increasingly important component of global economic and trade relations. At the same time, competition in global agricultural trade is shifting from traditional price-based competition to quality-based competition centered on safety, traceability, and sustainability. Consequently, agricultural export quality (AEQ) has become a key determinant of a country’s competitiveness in the global agricultural market. As one of the world’s leading agricultural producers, China has consistently ranked among the top countries in terms of agricultural export volume. In the first eleven months of 2025, China’s agricultural exports reached 670.21 billion yuan, representing a year-on-year increase of 2%, with more than 830 export categories covering over 220 countries and regions. Despite this considerable scale, China still lacks strong core competitiveness in AEQ. Therefore, improving AEQ has become a critical issue for promoting the high-quality development of China’s agricultural sector.

Agricultural digitalization refers to the extensive application of modern information technologies, such as the Internet of Things (IoT), big data, artificial intelligence (AI), and blockchain, to empower and transform the entire agricultural value chain. It has emerged as a key strategy for enhancing agricultural competitiveness, ensuring food security, and promoting sustainable development. As a traditional component of China’s export trade, agricultural exports urgently require a comprehensive digital and intelligent transformation. The integration of digital and intelligent technologies into agricultural production can cultivate new forms of productive capacity and shift agricultural development from a quantity-driven model to a quality-driven model, thereby providing a practical pathway for improving AEQ.

Globally, agricultural digitalization has become closely associated with improvements in AEQ and demonstrates strong empirical correlations. At the international level, the European Union’s Farm to Fork Strategy identifies digitalization as a key instrument for building a sustainable food system and enhancing supply chain transparency and traceability. Similarly, the Food and Agriculture Organization of the United Nations highlights in its FAO Strategic Framework 2020–2030 the transformative potential of digital agriculture for increasing productivity and strengthening food security and nutrition. Empirical evidence further supports these trends. Among Organisation for Economic Co-operation and Development (OECD) countries, the average adoption rate of precision agriculture technologies increased by approximately 25% between 2019 and 2023. As a result, agricultural products from these countries have gained price premiums in international markets due to stable quality and stronger compliance with international standards.

In summary, driven by both strong policy support and inherent market demand, agricultural digitalization has evolved from a simple technological upgrade into a key engine reshaping the international competitiveness of agricultural products, particularly in terms of export quality. However, the transformation process remains uneven across regions, industries, and stakeholders. The specific mechanisms through which agricultural digitalization affects AEQ, the methods for measuring its effects, and the policy pathways for optimizing this relationship remain insufficiently explored. This study therefore aims to systematically examine the intrinsic relationship between agricultural digitalization and AEQ improvement. By constructing evaluation indicator systems and measuring both agricultural digitalization and AEQ, this paper empirically analyzes their underlying relationships and operational pathways, providing theoretical foundations and policy references for accelerating agricultural digital transformation, enhancing AEQ, and promoting the high-quality development of agricultural trade.

Research on agricultural digitalization has mainly focused on the following aspects. First, studies examine the conceptual connotation of agricultural digitalization. As an integrated concept, the systematic definition of agricultural digitalization matured in the late 2010s. Laurens Klerkx et al. [1] were among the first to systematically review and differentiate related concepts such as digital agriculture, smart agriculture, Agriculture 4.0, and digital transformation. Their study emphasizes that agricultural digitalization goes beyond simple technology adoption; rather, it represents a profound transformation involving technological, organizational, business, and social changes. They also proposed a multidimensional research agenda covering technology, networks, business models, institutions, and ethical considerations. Around the same period, Sarah Rotz et al. [2] further clarified the concept by defining agricultural digitalization as a socio-technical process in which digital technologies, data, and connectivity fundamentally transform production practices, value chains, and business models. This definition has been widely cited due to its clear emphasis on socio-technical transformation. Simon Fielke et al. [3] further expanded the concept by examining the digital restructuring of agricultural knowledge and advisory networks. Their research highlights that agricultural digitalization extends far beyond on-farm technological applications, fundamentally transforming the ecosystem of knowledge generation, validation, dissemination, and extension services. In this context, traditional expert-led extension systems are gradually being replaced by decentralized, data-driven, and platform-mediated networks.

Second, existing studies examine the measurement and indicator systems used to evaluate agricultural digitalization. Given the complexity and multidimensional nature of the concept, most scholars construct composite indicator systems from multiple perspectives. Regarding measurement methods, many studies adopt objective weighting approaches, such as the entropy weight method, to construct evaluation systems (e.g., Mehrotra et al. [4]; Meng and Li [5]). A smaller number of studies employ quasi-experimental methods such as the Difference-in-Differences (DID) approach (e.g., Pierpaoli et al. [6]; Zheng et al. [7]), while others utilize methods such as Data Envelopment Analysis (DEA) to analyze the efficiency of digital agricultural development (Zhang et al. [8]). In terms of indicator selection, most existing studies construct evaluation frameworks based on factors such as the development environment for agricultural digitalization, digital infrastructure, technological and human capital resources, digital agricultural industries, and the economic outcomes of digital transformation (e.g., Wang et al. [9]; Zhou et al. [10]).

Third, scholars have explored the functional pathways through which agricultural digitalization influences agricultural development. Chang [11] and Guo et al. [12] demonstrate that agricultural digitalization significantly reduces agricultural carbon emissions, and Chang further finds that such effects also generate negative spatial spillovers in neighboring regions. Dong and Xu [13] provide empirical evidence showing that agricultural digitalization strongly promotes the development of a modern agricultural system and contributes to the construction of a strong agricultural nation. Li et al. [14] further examine the relationship between agricultural digitalization and agricultural green total factor productivity, finding that digitalization significantly improves green productivity while exhibiting substantial regional heterogeneity.

Research on agricultural export quality (AEQ) primarily focuses on two aspects. First, studies investigate methods for measuring AEQ. Most scholars adopt the approach proposed by Ricardo Hausmann et al. [15], which measures export quality in terms of technological complexity. This study also adopts this approach. Alternatively, Fontagné et al. [16] use the proportion of exports to high-income countries or the average income level of export destinations as proxy indicators of product quality, based on the assumption that higher-quality goods are more likely to be exported to wealthier markets. Crivelli and Gröschl [17] measure quality using indicators such as the number of certifications complying with international standards (e.g., ISO or SPS agreements) or export inspection pass rates.

Second, numerous studies examine the determinants of AEQ. Existing literature suggests that factors such as market demand, trade policies, and technological development can influence export quality. Juan Carlos Hallak [18] finds that demand from high-income markets for higher-quality products encourages exporting countries to upgrade product quality. Pablo Fajgelbaum [19] argues that trade liberalization, particularly tariff reductions, intensifies competition among firms in high-income markets and encourages quality upgrading. Fernandes et al. [20] and Zhou and Zhang et al. [21] provide empirical evidence that agricultural digitalization can significantly promote improvements in AEQ.

Several studies specifically examine the relationship between agricultural digitalization and AEQ. Liu and Li [22] focus on rural e-commerce as a key form of agricultural digitalization and analyze its impact on AEQ upgrading. Their results show that the development of e-commerce significantly increases the average export unit price of agricultural products, particularly for high-value-added products. Wang and Li [23] directly investigate the impact of agricultural digitalization on AEQ upgrading in China and find that digitalization significantly promotes export quality improvement through mechanisms such as productivity enhancement, product innovation, and more efficient resource allocation. Other studies provide indirect evidence supporting this relationship. For example, Lendle et al. [24] show that online platforms such as eBay reduce trade costs by improving trust and simplifying transactions, thereby facilitating the trade of complex and high-quality goods, including credence goods such as agricultural products. Bao and Chen [25] demonstrate that proactive adoption of international standards such as GlobalG.A.P. is crucial for developing countries seeking to upgrade export quality, and that digital agricultural technologies, such as digital production management systems and sensor networks, are essential tools for achieving and verifying compliance with these standards.

Synthesizing the existing literature reveals two key gaps. First, most studies examine the conceptual definitions, development status, pathways, and influencing factors of agricultural digitalization or AEQ separately, while relatively few studies empirically analyze the direct relationship between the two. Second, given the rapidly evolving international agricultural trade environment and the demands of national development, further research is needed to explore the deeper connections between agricultural digitalization and AEQ and to propose effective policy recommendations based on these relationships.

Building upon the existing literature, this paper makes three main contributions. First, it constructs a comprehensive evaluation indicator system for agricultural digitalization and empirically examines whether and how agricultural digitalization affects AEQ, thereby extending and enriching the existing research. Second, using provincial panel data, this study measures both agricultural digitalization and AEQ and conducts multidimensional heterogeneity analyses to explore the varying effects of agricultural digitalization under different conditions. It further incorporates moderating variables to investigate the roles of policy support intensity and market openness in shaping this relationship. Third, based on recent national policy priorities and agricultural development strategies, the paper proposes policy recommendations for promoting agricultural digital transformation and improving AEQ, thereby contributing to the refinement of the existing theoretical framework.

## 2. Mechanism analysis and research hypotheses

### 2.1 Direct impact of agricultural digitalization on AEQ

Agricultural digitalization enhances AEQ by deeply integrating technologies such as the Internet of Things, big data, and blockchain into production, management, and circulation processes. This integration not only enables precise control and standardized management throughout the agricultural production chain, but also proactively addresses the increasingly stringent international demands for quality and safety through tools like intelligent sorting and traceability systems. It effectively reduces supply chain losses, overcomes the trust deficit in international trade, and ultimately ensures that agricultural products systematically meet and exceed the export standards of target markets, building a sustainable quality advantage in global competition.

First, agricultural digitalization directly reduces information asymmetry regarding agricultural product quality. By applying technologies like IoT and blockchain, a complete, immutable digital footprint is created for each exported agricultural product. This process establishes a low-cost, verifiable quality information transmission system between producers and overseas consumers, directly addressing a core challenge in international trade: the lack of trust. Exporters can demonstrate to buyers at near-zero marginal cost that their products comply with specific standards at every stage from cultivation and processing to storage and this way can reduce significantly high transaction costs associated with inquiries, inspections, and disputes caused by quality opacity. This reduction in information friction itself directly enhances the “credibility quality” of export products, strengthening their bargaining power and market access in high-end segments.

Furthermore, digital technologies are directly incorporated into the production function as new forms of capital goods, fundamentally transforming the process through which quality is shaped. For instance, intelligent greenhouse control systems and precision irrigation facilities essentially shift decision-making from experience-based judgments to real-time data-driven insights, while converting manual operations into automated execution. This substitution yields two primary economic outcomes: first, it significantly reduces quality fluctuations caused by human error, thereby enhancing the standardization and predictability of outputs; second, through precise control over variable inputs such as water, fertilizers, and pesticides, it optimizes the quality attributes of the final products such as uniformity, taste, or safety, while simultaneously lowering marginal costs. This effect is direct, stemming not from the upgrading of other production factors but from the immediate improvement in technical efficiency brought about by digital capital goods within the production process itself.

Therefore, it can be concluded that agricultural digitalization can improve AEQ by effectively reducing information asymmetry regarding agricultural product quality and optimizing production processes. Accordingly, this study proposes Hypothesis 1:

*H*_1_: Agricultural digitalization can effectively improve AEQ.

### 2.2 Indirect impact of agricultural digitalization on AEQ

The deeper impact of agricultural digitalization lies in its ability to indirectly and systematically enhance export quality by altering key factor endowments and resource allocation efficiency within the industry. This process can be understood as digital technology generating spillover effects that permeate and enhance the productivity of other factors of production, ultimately leading to improvements in quality. Building on this, and referencing studies by Tong et al. [26], Tian et al. [27] and Qiu and MOHAMAD [28], this paper investigates the mechanisms through which agricultural digitalization affects AEQ from three dimensions: agricultural technological progress, agricultural human capital, and agricultural logistics efficiency.

#### 2.2.1 Agricultural technological progress.

Agricultural technological progress is a core mediating channel through which agricultural digitalization affects export quality. Digital technology itself is not synonymous with agricultural technology, but by providing low-cost data acquisition and processing capabilities, it significantly facilitates the dissemination, adaptation, and iteration of knowledge and technology within production processes. This leads to an increase in total factor productivity, ultimately reflected in product quality upgrading.

Specifically, agricultural digitalization reshapes the patterns of technology R&D and diffusion. On the R&D front, simulation and analysis based on big data accelerate processes like crop breeding and feed formulation, reducing experimental costs and enabling the faster emergence of varieties and technologies better suited to specific market and quality demands. On the application front, the effectiveness of precision agriculture technologies based on satellite remote sensing and sensor networks is highly dependent on digital infrastructure. This allows advanced agronomic knowledge, previously confined to laboratories or large farms, to be codified into standardized data models and operational instructions delivered to a wide range of farmers via smart machinery or mobile applications. This “technology empowerment” enables the large-scale, standardized implementation of quality-enhancing agronomic practices, such as scientific soil management and integrated pest management.

Therefore, by promoting technological progress and diffusion, agricultural digitalization indirectly yet profoundly shifts the agricultural production possibility frontier. It makes the technical knowledge required for producing higher-quality products more accessible and actionable, thereby basing export quality improvement on sustained technological advancement rather than relying solely on end-point inspection.

#### 2.2.2 Agricultural human capital.

Human capital is a crucial factor for absorbing and applying new technologies. The indirect impact of agricultural digitalization on export quality can only be fully realized through the mediation of enhanced labor skills and knowledge levels. This process aligns with the technology-skill complementarity view in new growth theory.

On one hand, the application of digital tools creates new demand for high-skilled labor. Operating smart agricultural machinery, analyzing agricultural big data, and managing e-commerce platforms require workers with composite skills. This attracts individuals with higher educational backgrounds and digital literacy into the agricultural sector, structurally elevating the industry’s overall human capital level. This segment of the workforce can more effectively translate the potential of digital technologies into management decisions and quality control practices.

On the other hand, and more importantly, digital platforms themselves become effective vehicles for investing in the knowledge of the existing workforce. Mobile applications, online training videos, and interactive expert systems provide immediate production guidance and quality standard information to millions of dispersed farmers at very low marginal cost. While this type of widespread skills training may not directly cultivate advanced experts, it can rapidly and broadly enhance basic producers’ quality awareness and standardized operational capabilities, narrowing the gap between production practices and international market requirements.

Thus, human capital plays a key mediating role: agricultural digitalization creates both the demand and the tools for skills enhancement, and the corresponding improvement in human capital ensures that technologies are effectively adopted and correctly utilized, thereby translating digital investments into stable, reliable, high-quality output. Without concomitant human capital upgrading, the return on investment in digital equipment would be significantly diminished.

#### 2.2.3 Agricultural logistics efficiency.

Agricultural logistics efficiency is critical for determining whether agricultural products can retain their inherent quality upon arrival in international markets. agricultural digitalization significantly reduces value loss in the circulation phase by enhancing the efficiency of resource allocation and the controllability of processes within the logistics system, thereby indirectly safeguarding and elevating the final delivered export quality.

From an economic standpoint, the core challenge in agricultural logistics lies in coordinating the complex allocation of temporal and spatial resources while managing the substantial associated risks. The mediating role of digitalization is evident in three key areas: First, the optimization of resource allocation through real-time data. Utilizing big data to forecast market demand, analyze global port congestion, and deploying algorithms to plan optimal shipping routes and warehouse schedules drastically cuts down on unproductive idle time for goods during transit and storage. Time saved directly correlates with better preservation of product freshness and nutritional value.

Second, quality risks are mitigated through IoT monitoring. Deploying sensors in refrigerated containers for temperature and humidity monitoring is equivalent to purchasing “end-to-end quality insurance” for perishable goods. Once environmental parameters deviate from safe ranges, the system can trigger automatic alerts or even adjustments. This directly reduces the probability of quality deterioration or total loss of entire shipments due to environmental factors. This enhancement of risk management lowers the operational risks and contractual costs faced by both exporters and importers.

Third, the transparency of logistics information improves the overall coordination efficiency of the supply chain. When production information is seamlessly integrated with real-time logistics data, more precise coordination among exporters, shipping companies, and importers can be achieved. This reduces procedural frictions such as waiting and repeated inspections, enabling products to be delivered in optimal condition and within expected timeframes. Consequently, reliable expectations regarding quality consistency are established among overseas consumers. Therefore, the enhancement of agricultural logistics efficiency serves as a key intermediary link through which agricultural digitalization reduces trade costs and ensures the preservation of product value during spatial transfer.

In summary, agricultural technological progress, agricultural human capital, and agricultural logistics efficiency are all significant pathways through which agricultural digitalization promotes AEQ improvement. Therefore, Hypothesis 2 is proposed:

H2: Agricultural technological progress, agricultural human capital, and agricultural logistics efficiency are effective indirect pathways through which agricultural digitalization drives the improvement of AEQ.

### 2.3 Analysis of the moderating mechanisms of policy support and market openness

#### 2.3.1 The moderating effect of policy support.

Government policy support serves as a critical enabler and risk mitigator in the process of agricultural digitalization. Its moderating effect is primarily manifested in three areas: resource provision, rule-making, and directional guidance.

Strong policy support can effectively lower the initial investment threshold and the cost of trial and error associated with agricultural digitalization. Direct government investment or subsidies for digital infrastructure, along with purchase subsidies for new smart agricultural machinery and IoT equipment, directly alleviate the capital constraints faced by agricultural operators, especially small and medium-sized entities. This enables a greater number of producers to integrate into the digital ecosystem, thereby expanding the scope of participants in quality improvement and broadening the overall impact. Conversely, a lack of policy support may confine agricultural digitalization to a limited number of large enterprises, significantly diminishing its inclusivity and overall industrial effect on quality enhancement.

Furthermore, clear policies and regulations provide institutional legitimacy and unified standards for digital quality traceability systems. Government-led establishment of national or industry standards for data collection, exchange, and privacy protection across the entire agricultural supply chain can dismantle transmission barriers created by disparate systems developed by different market players. This facilitates the seamless flow of traceability information within the supply chain, greatly enhancing the credibility and scale effects of the digital trust system. Simultaneously, by linking digital traceability with regulatory incentives such as market access, quality certification, and brand evaluation, policies create powerful external incentives for producers to adopt digital technologies for quality improvement, thereby moderating their behavioral motivation.

Finally, forward-looking industrial policies and strategic planning guide the quality orientation of digital investments. Policies that focus on supporting digital solutions for enhancing quality and promoting green production, rather than merely pursuing yield increases, directly channel technology R&D and application resources towards quality-focused domains. This positively moderates the direction and depth of the impact of agricultural digitalization on export quality.

#### 2.3.2 The moderating role of market openness.

Market openness moderates the quality-enhancing effect of agricultural digitalization through both motivational and pressurizing mechanisms by altering the competitive environment, technology accessibility, and demand standards.

A high level of market openness implies more intense international competition and more direct exposure to the demands of high-end markets. Faced with competitive pressure from high-quality global agricultural products, domestic exporters have a stronger intrinsic motivation to invest in agricultural digitalization to improve quality and achieve differentiation for survival and growth. Concurrently, an open market facilitates the direct introduction of advanced foreign agricultural digital technologies, intelligent equipment, and management concepts, accelerating domestic technology spillover and the learning process. This enhances the technological substance and application efficacy of the agricultural digitalization itself, strengthening its technical impetus for quality improvement.

More importantly, market openness exposes domestic producers directly to the stringent quality and safety standards set by mainstream international markets and large buyers. To enter these markets, compliance with increasingly complex non-price requirements regarding production process traceability, environmental sustainability, and labor standards becomes essential. This “standards-pull” effect from the demand side provides a clear, urgent, and high-standard application target for agricultural digitalization. It compels digital investments to serve not only productivity gains but also, and crucially, the goals of meeting international certifications and providing verifiable compliance evidence, thereby significantly strengthening the association between agricultural digitalization and export quality standard upgrading.

Conversely, in a context of lower market openness, where markets are more protected, competitive pressure is insufficient, and demand standards may remain at a relatively low level for an extended period, the primary driver for agricultural digitalization may stem from internal cost-saving motives rather than from a pursuit of quality leapfrogging. This would weaken its role in pursuing and shaping high-end export quality. Therefore, through the introduction of external competition and high-standard demands, market openness serves as a vital “pressure test” and “direction calibrator” moderating the full unleashing and utilization of agricultural digitalization’s quality-upgrading potential.

Based on the above analysis, this study proposes Hypothesis 3

H3: Policy support and market openness play moderating roles in the process through which agricultural digitalization affects AEQ.

## 3. Model specification and variable definitions

### 3.1 Model construction

#### 3.1.1 Baseline regression.

Based on the theoretical analysis and hypotheses presented earlier, to empirically examine the impact of agricultural digitalization on AEQ, this paper constructs the following set of regression models:


$$lnAEQ_{it}=\alpha_{\text{0}}\text{+}\alpha_{\text{1}}AD_{it}\text{+}\alpha_{\text{2}}Mediator_{it}\text{+}\alpha_{\text{3}}Moderator_{it}\text{+}\alpha_{\text{4}}Control_{it}\text{+}\delta_{i}\text{+}\delta_{t}\text{+}\varepsilon_{it}$$ (1)

In the equation: AEQ represents the dependent variable, AEQ; AD represents the explanatory variable, agricultural digitalization; Mediator represents the mediator variable; Moderator represents the moderator variable; Control represents the control variables; $\delta_{i}$, $\delta_{t}$ denote province and time fixed effects, respectively; εit denotes the stochastic error term.

#### 3.1.2 Mediation effect.

To test H2, the following mechanism test model is constructed to examine the impact mechanism of agricultural digitalization on AEQ. Drawing on the research of Judd and Kenny [29], as well as Baron and Kenny [30], this study establishes the following mediation model:


$$Mediator_{it}=\beta_{\text{0}}+\beta_{\text{1}}AD_{it}+\beta_{\text{2}}Control_{it}+\delta_{i}+\delta_{t}+\mu_{it}$$ (2)

$$lnAEQ_{it}=\gamma_{\text{0}}\text{+}\gamma_{\text{1}}AD_{it}\text{+}\gamma_{\text{2}}Mediator_{it}\text{+}\gamma_{\text{3}}Control_{it}\text{+}\delta_{i}\text{+}\delta_{t}\text{+}\nu_{it}$$ (3)

In the equation:Mediatorrepresents the mediator variables, namely agricultural technological progress, agricultural human capital, and agricultural logistics efficiency.

#### 3.1.3 Moderating role.

To test H3, drawing on relevant research by Aiken and West [31], this paper constructs a moderating effect model to verify the moderating role played by policy support and market openness in the process through which agricultural digitalization affects AEQ. The model is specified as follows:


$$\begin{array}{c} \begin{array}{cc} InAEQ_{it}=\;\;\;\; & \phi_{0}+\phi_{1}AD_{it}+\phi_{2}Moderator_{it}+\phi_{3}AD_{it}\times Moderator_{it}\\ & +\;\phi_{4}Control_{it}+\delta_{i}+\delta_{t}+\varphi_{it} \end{array}\mathrm{\backslash end\{aligned\}} \end{array}$$ (4)

In the equation:Moderatorrepresents the moderator variables, namely policy support and market openness.

#### 3.1.3 Moderating role.

To test H3, drawing on relevant research by Aiken and West [31], this paper constructs a moderating effect model to verify the moderating role played by policy support and market openness in the process through which agricultural digitalization affects AEQ. The model is specified as follows:


$$\begin{array}{c} \begin{array}{cc} InAEQ_{it}=\;\;\;\; & \phi_{0}+\phi_{1}AD_{it}+\phi_{2}Moderator_{it}+\phi_{3}AD_{it}\times Moderator_{it}\\ & +\;\phi_{4}Control_{it}+\delta_{i}+\delta_{t}+\varphi_{it} \end{array} \end{array}$$ (5)

In the equation: Moderator represents the moderator variables, namely policy support and market openness.

### 3.2 Variable selection

#### 3.2.1 Explained variable.

The explained variable in this paper is AEQ (*AEQ*). Drawing on the methods of Hausmann et al. [15] and Lin et al. [32], the AEQ of each province is measured using the approach of technical complexity.

#### 3.2.2 Explanatory variable.

The explanatory variable in this paper is agricultural digitalization (*AD*), represented by the level of agricultural digitalization. Based on the research of Hua et al. [33] and Wang et al. [34], this study measures it using the entropy method, grounded in three first-level indicators (agricultural digital infrastructure, digital production application, digital industry connection) and nine second-level indicators. The specific indicator system is shown in Table 1.

**Table 1:** Indicator system for the level of agricultural digitalization.

Level 1 Indicators	Level 2 Indicators	Indicator explanation	Attribute
agricultural digital infrastructure	Rural internet penetration rate	Rural broadband access subscribers/ number of rural households	+
	Rural smartphone penetration rate	Average number of mobile phones owned per 100 rural households annually	+
	Agricultural meteorological observation stations	Number of agricultural meteorological observation stations	+
digital production application	Agricultural machinery total power	Total power of various power machinery in agriculture, forestry, animal husbandry, and fishery	+
	Proportion of smart agriculture planting area	Digitally managed planting area/ total regional planting area	+
	Agricultural product traceability system coverage rate	Proportion of agricultural product categories connected to the traceability platform	+
digital industry connection	Agricultural product digital transactions	Online retail sales of agricultural products	+
	Digital agriculture and rural innovation bases	Number of Taobao villages	+
	Rural digital payment scale	Digital inclusive finance Index	+

#### 3.2.3 Control variables.

Based on the research focus and agricultural orientation of this paper, nine control variables have been selected. The specific indicator interpretations and measurement methods are as follows: (1) the level of economic development (*GDP*), which is measured by the annual per capita gross regional product of each province and then logarithmically processed to reflect its impact more precisely; (2) the market size (*Scale*), represented by the annual total population of each province and also logarithmically processed; (3) the efficiency of agricultural production (*RCA*), expressed as the annual per capita agricultural increase of each province and logarithmically processed as well to eliminate the impact of data scale; (4) the industrial structure (*Industry*), depicted by the ratio of the output value of the primary industry to the Gross Regional Product (GRP); (5) the foreign investment (*FDI*), measured by the ratio of the actual use of FDI to the Gross Regional Product (GRP); (6) the agricultural Climate Conditions (*Climate*), measured by the main crops’suitable planting index (dimensionless); (7) the land scale level (*LandScale*), measured by average operating area of family farms; (8) the agricultural financial support (*FinSupport*), measured by ratio of agricultural loan balance to total regional loan balance; (9) the trade policy environment (*TradeEnv*), measured by proportion of trade volume with RCEP member countries.

#### 3.2.4 Mediating variables.

The mediating variables in this study are as follows:the agricultural technological progress (*Tech*), measured by ratio of agricultural R&D investment to the total output value of agriculture, forestry, animal husbandry and fishery; the agricultural human capital (*Human*), measured by proportion of rural labor force with senior high school or above education; the agricultural logistics efficiency (*Logis*), measured by number of agricultural cold chain logistics enterprises divided by agricultural output value.

#### 3.2.5 Moderator variables.

The moderator variables in this study are as follows:the policy support (*PolicySupport*), measured by comprehensive index of agricultural support policies (including subsidies, tax incentives, etc.), estimated by the entropy method; the aarket openness (*MarketOpenness*), measured by ratio of total import and export volume to regional GDP (logarithmically processed). The details of indicator system is shown in Table 2.

**Table 2:** Indicator system.

Variable type	Variable name	Variable definition	Measurement method
Dependent variable	*AEQ*	AEQ	Export technological complexity calculated via the Hausmann method
Core independent variable	*AD*	Agricultural digitalization	Constructed from 3 first-level indicators (agricultural digital infrastructure, digital production application, digital industry connection) and 9 secondary indicators, estimated by the entropy method
Mediation variable	*Tech*	Agricultural technological progress	Ratio of agricultural R&D investment to the total output value of agriculture, forestry, animal husbandry and fishery
	*Human*	Agricultural human capital	Proportion of rural labor force with senior high school or above education
	*Logis*	Agricultural logistics efficiency	Number of agricultural cold chain logistics enterprises divided by agricultural output value
Control variable	*GDP*	Economic development level	Per capita regional GDP (logarithmically processed)
	*Scale*	Market size	Total regional population (logarithmically processed)
	*RCA*	Agricultural production efficiency	Per capita agricultural added value (logarithmically processed)
	*Industry*	Industrial structure	Ratio of primary industry output value to regional GDP
	*FDI*	Foreign direct investment	Ratio of actually utilized FDI to regional GDP
	*Climate*	Agricultural climate conditions	Main crops’ suitable planting index (dimensionless)
	*LandScale*	Land scale level	Average operating area of family farms (mu)
	*FinSupport*	Agricultural financial support	Ratio of agricultural loan balance to total regional loan balance
	*TradeEnv*	Trade policy environment	Proportion of trade volume with RCEP member countries
Moderation variable	*PolicySupport*	Policy support	Comprehensive index of agricultural support policies (including subsidies, tax incentives, etc.), estimated by the entropy method
	*MarketOpenness*	Market openness	Ratio of total import and export volume to regional GDP (logarithmically processed)
Instrument variable	*IV*_1_ *(Lagged AD)*	Lagged agricultural digitalization	Lagged 1-period value of AD (used as instrument variable for 2SLS)
	*IV*_2_ *(Bartik Index)*	Bartik instrument variable	Product of national agricultural digitalization growth rate and provincial initial agricultural digitalization level

### 3.3 Data sources and descriptive statistics of variables

The data on agricultural export quality (AEQ) used in this study are obtained from the National Research Network and the United Nations Trade Database. Data on agricultural digitalization are collected from the China Statistical Yearbook, the Financial Research Center of Peking University, and the China County Statistical Yearbook. Data for the remaining variables are sourced from the National Bureau of Statistics of China and the statistical bureaus of individual provinces. The descriptive statistics of each variable are presented in Table 3.

**Table 3:** Descriptive statistics.

Variables	Obs.	Mean	Std. Dev.	Min.	Max.
*AEQ*	816	8.326	0.518	6.982	9.875
*AD*	816	2.158	0.634	0.892	4.321
*Tech*	816	3.624	0.875	1.563	5.987
*Human*	816	2.873	0.742	1.235	4.968
*Logis*	816	3.215	0.913	1.452	5.734
*GDP*	816	10.236	1.568	7.892	13.654
*Scale*	816	8.562	1.231	6.345	11.287
*RCA*	816	4.328	0.987	2.156	6.892
*Industry*	816	0.185	0.064	0.072	0.396
*FDI*	816	0.052	0.021	0.013	0.128
*Climate*	816	0.683	0.157	0.321	0.945
*LandScale*	816	5.217	1.342	2.876	8.963
*FinSupport*	816	0.086	0.032	0.025	0.197
*TradeEnv*	816	0.235	0.089	0.092	0.468
*PolicySupport*	816	1.872	0.563	0.785	3.421
*MarketOpenness*	816	2.015	0.624	0.896	3.875

## 4. Empirical analysis

### 4.1 Baseline regression

Table 4 reports the baseline regression results for the impact of agricultural digitalization on agricultural export quality (AEQ). Columns (1)-(4) sequentially incorporate control variables and fixed effects to examine the robustness of the core relationship. The coefficient of *AD* remains significantly positive at the 1% level across all specifications, declining slightly from 0.057 in the univariate regression to 0.045 in the full-specification model (Column 4). This finding indicates that agricultural digitalization significantly promotes improvements in AEQ, and the positive effect remains robust after controlling for additional factors and both individual and time fixed effects. Among the control variables, GDP and RCA are significantly positive at the 1% level, suggesting that regional economic development and enhanced agricultural productivity contribute to improvements in export quality. The coefficient of Industry is significantly negative, indicating that a higher share of the primary industry may hinder the upgrading of AEQ. In addition, *FDI*, *Climate*, *LandScale*, *FinSupport*, and *TradeEnv* all exert positive effects on AEQ, which is consistent with theoretical expectations. The adjusted *R*^*2*^ increases from 0.215 to 0.513, indicating that the model possesses strong explanatory power. Overall, these results provide empirical support for Hypothesis *H*_1_.

**Table 4:** Benchmark regression results.

Variables	(1) *AEQ*	(2) *AEQ*	(3) *AEQ*	(4) *AEQ*
*AD*	0.057***	0.053**	0.048***	0.045***
	(0.005)	(0.006)	(0.006)	(0.005)
*GDP*		0.032***	0.029**	0.027***
		(0.009)	(0.008)	(0.007)
*Scale*		0.018**	0.016**	0.015**
		(0.008)	(0.007)	(0.006)
*RCA*		0.035***	0.031**	0.029***
		(0.007)	(0.006)	(0.006)
*Industry*		−0.124***	−0.117**	−0.112***
		(0.028)	(0.026)	(0.025)
*FDI*		0.021***	0.019***	0.018**
		(0.004)	(0.004)	(0.003)
*Climate*			0.014***	0.013***
			(0.003)	(0.003)
*LandScale*			0.020**	0.018**
			(0.004)	(0.003)
*FinSupport*			0.011*	0.010***
			(0.003)	(0.002)
*TradeEnv*			0.016***	0.015**
			(0.004)	(0.003)
*_cons*	8.253***	8.017***	7.924***	7.941***
	(0.028)	(0.056)	(0.063)	(0.059)
Province Fixed Effects	No	No	No	Yes
Year Fixed Effects	No	No	No	Yes
Obs.	816	816	816	816
R²	0.215	0.387	0.462	0.513

***, **, and * indicate significance at the 1%, 5%, and 10% levels, respectively; standard errors are reported in parentheses.

### 4.2 Endogeneity tests

To address potential endogeneity concerns, such as reverse causality and omitted variable bias, Table 5 employs three approaches: the two-stage least squares (2SLS) method, the Heckman two-step procedure, and the Bartik instrumental variable (IV) approach. For the 2SLS estimation (Columns 1–2), the lagged value of *AD* (IV_1_) is used as the instrumental variable. The first-stage results show that IV_1_ is significantly and positively correlated with *AD* (coefficient = 0.782). In addition, the Cragg-Donald Wald F-statistic (231.56) is substantially higher than the Stock-Yogo critical value, thereby rejecting the weak-instrument hypothesis. The second-stage estimate indicates that the coefficient of *AD* is 0.058 and statistically significant at the 1% level, confirming the positive effect.

**Table 5:** Endogeneity test results.

Variables	(1) 2SLS (First Stage)	(2) 2SLS (Second Stage)	(3) Heckman (Selection Equation)	(4) Heckman (Outcome Equation)	(5) Bartik IV (First Stage)	(6) Bartik IV (Second Stage)
*AD*		0.058***		0.052***		0.061***
		(0.008)		(0.009)		(0.009)
*IV*_1_ *(Lagged AD)*	0.782***					
	(0.041)					
*IV*_2_ *(Bartik Index)*					0.693***	
					(0.038)	
*Digital Infrastructure Access*			0.315***			
			(0.032)			
*GDP*	0.042***	0.029**	0.051***	0.028**	0.038**	0.030***
	(0.010)	(0.008)	(0.012)	(0.008)	(0.010)	(0.008)
*Scale*	0.025**	0.016**	0.032***	0.015**	0.023**	0.017**
	(0.009)	(0.007)	(0.010)	(0.007)	(0.009)	(0.007)
*RCA*	0.041***	0.027**	0.048***	0.026**	0.039**	0.028***
	(0.008)	(0.007)	(0.010)	(0.007)	(0.008)	(0.007)
*Industry*	−0.182***	−0.125***	−0.203**	−0.119***	−0.175**	−0.128***
	(0.035)	(0.026)	(0.038)	(0.025)	(0.034)	(0.026)
*FDI*	0.028***	0.016**	0.033***	0.015**	0.026***	0.017***
	(0.005)	(0.004)	(0.006)	(0.004)	(0.005)	(0.004)
*Climate*	0.019***	0.011***	0.023***	0.010***	0.018***	0.012***
	(0.004)	(0.003)	(0.005)	(0.003)	(0.004)	(0.003)
*LandScale*	0.027***	0.015***	0.031**	0.014***	0.025**	0.016***
	(0.005)	(0.004)	(0.006)	(0.004)	(0.005)	(0.004)
*FinSupport*	0.016***	0.008**	0.019***	0.007**	0.015***	0.009**
	(0.003)	(0.003)	(0.004)	(0.003)	(0.003)	(0.003)
*TradeEnv*	0.023***	0.013***	0.027**	0.012***	0.022***	0.014***
	(0.004)	(0.003)	(0.005)	(0.003)	(0.004)	(0.003)
*_cons*	6.892***	7.528***	5.936***	7.614***	7.015***	7.483***
	(0.124)	(0.065)	(0.141)	(0.063)	(0.118)	(0.064)
Province FE	Yes	Yes	Yes	Yes	Yes	Yes
Year FE	Yes	Yes	Yes	Yes	Yes	Yes
Obs.	744	744	816	816	744	744
R²	0.638	0.542	0.685	0.537	0.615	0.551
F-Statistic	89.37***	76.52***			82.45***	79.13***
Cragg-Donald Wald F-Stat	231.56				198.74	
Stock-Yogo Critical Value (10%)	16.38				16.38	
Lambda (Heckman)			0.083***	(0.021)		
rho (Heckman)			0.315***	(0.068)		

***, **, and * indicate significance at the 1%, 5%, and 10% levels, respectively; standard errors are reported in parentheses.

The Heckman two-step estimation (Columns 3–4) uses digital infrastructure accessibility as the selection variable. The statistically significant Lambda coefficient (0.083) suggests the presence of sample selection bias. After correcting for this bias, the coefficient of *AD* in the outcome equation (0.052) remains significantly positive.

The Bartik IV approach (Columns 5–6) produces consistent results. The estimated coefficient of *AD* is 0.061 and remains significant at the 1% level. Overall, the results obtained from the three endogeneity treatments consistently demonstrate that the positive impact of agricultural digitalization on AEQ is robust and not driven by endogeneity concerns.

### 4.3 Robustness tests

Table 6 verifies the robustness of the benchmark results through multiple methods. Columns (2)-(3) replace the dependent variable with export competitiveness and markup, and the coefficients of *AD* are 0.062 and 0.057 respectively, both significant at the 1% level. Column (4) adopts the Tobit model to address potential censoring bias, and the core coefficient (0.042) remains significantly positive. Column (5) conducts 1% bilateral winsorization to eliminate the impact of outliers, and the results are consistent with the benchmark regression.These robustness tests from different perspectives confirm that the core conclusion of this study is reliable and not affected by variable measurement, model setting, sample selection.

**Table 6:** Robustness test results.

Variables	(2) Core Var. Replace (Export Competitiveness)	(3) Core Var. Replace (Markup)	(4) Model Replace (Tobit)	(5) Sample Replace (1% Winsorize)
*AD*	0.062***	0.057***	0.042***	0.043***
	(0.007)	(0.006)	(0.005)	(0.005)
*GDP*	0.035***	0.031***	0.025**	0.026***
	(0.008)	(0.007)	(0.006)	(0.007)
*Scale*	0.021**	0.018**	0.013**	0.014**
	(0.008)	(0.007)	(0.006)	(0.006)
*RCA*	0.036***	0.033***	0.027**	0.028***
	(0.007)	(0.006)	(0.006)	(0.006)
*Industry*	−0.143***	−0.131***	−0.105***	−0.108***
	(0.029)	(0.027)	(0.024)	(0.024)
*FDI*	0.024***	0.021***	0.017***	0.017***
	(0.004)	(0.003)	(0.003)	(0.003)
*Climate*	0.017**	0.015***	0.012***	0.012***
	(0.004)	(0.003)	(0.003)	(0.003)
*LandScale*	0.023***	0.020***	0.016**	0.017***
	(0.004)	(0.003)	(0.003)	(0.003)
*FinSupport*	0.013***	0.011***	0.009**	0.009**
	(0.003)	(0.002)	(0.002)	(0.002)
*TradeEnv*	0.019***	0.017***	0.014***	0.014***
	(0.004)	(0.003)	(0.003)	(0.003)
*_cons*	7.628***	7.735***	7.986***	7.953***
	(0.071)	(0.065)	(0.061)	(0.060)
Province FE	Yes	Yes	Yes	Yes
Year FE	Yes	Yes	Yes	Yes
Obs.	816	816	816	792
R²	0.567	0.542		0.508
Pseudo R²			0.486	
Log Likelihood			589.32	
Sigma (Tobit)			0.127***	

***, **, and * indicate significance at the 1%, 5%, and 10% levels, respectively; standard errors are reported in parentheses.

### 4.4 Mediation effect tests

Table 7 presents the mediation effect test results, investigating the indirect transmission mechanisms of agricultural digitalization on AEQ through the agricultural technological progress (*Tech)*, the agricultural human capital (*Human*), and the agricultural logistics efficiency (*Logis*). Columns (1)-(3) show that *AD* has significantly positive impacts on the three mediator variables at the 1% level, with coefficients of 0.216, 0.189, and 0.175 respectively. This confirms that agricultural digitalization can effectively promote agricultural technological progress, optimize human capital allocation, and improve logistics efficiency.

**Table 7:** Mediation effect test results.

Variables	*(1) Tech*	*(2) Human*	*(3) Logis*	*(4) AEQ*	*(5) AEQ*	*(6) AEQ*
*AD*	0.216**	0.189***	0.175**	0.034**	0.036***	0.037***
	(0.023)	(0.020)	(0.019)	(0.006)	(0.006)	(0.006)
*Tech*				0.028***		
				(0.003)		
*Human*					0.024***	
					(0.003)	
*Logis*						0.022***
						(0.003)
*GDP*	0.152***	0.136***	0.129***	0.022**	0.023***	0.024***
	(0.018)	(0.016)	(0.015)	(0.007)	(0.007)	(0.007)
*Scale*	0.095**	0.087**	0.081**	0.012**	0.013**	0.013**
	(0.017)	(0.015)	(0.014)	(0.006)	(0.006)	(0.006)
*RCA*	0.173***	0.162***	0.155***	0.020***	0.021***	0.021***
	(0.015)	(0.014)	(0.013)	(0.006)	(0.006)	(0.006)
*Industry*	−0.315***	−0.297***	−0.286*	−0.098**	−0.101***	−0.103***
	(0.056)	(0.052)	(0.050)	(0.024)	(0.024)	(0.025)
*FDI*	0.106***	0.098***	0.092***	0.014***	0.015***	0.015***
	(0.009)	(0.008)	(0.008)	(0.003)	(0.003)	(0.003)
*Climate*	0.072***	0.068***	0.063***	0.009**	0.009**	0.010**
	(0.007)	(0.006)	(0.006)	(0.003)	(0.003)	(0.003)
*LandScale*	0.091***	0.085**	0.079***	0.014***	0.014***	0.015***
	(0.008)	(0.007)	(0.007)	(0.003)	(0.003)	(0.003)
*FinSupport*	0.065**	0.061**	0.057**	0.007*	0.007**	0.008**
	(0.007)	(0.006)	(0.006)	(0.002)	(0.002)	(0.002)
*TradeEnv*	0.083***	0.077***	0.072**	0.011***	0.012***	0.012***
	(0.009)	(0.008)	(0.008)	(0.003)	(0.003)	(0.003)
*_cons*	5.216***	5.438***	5.562***	7.615***	7.582***	7.569***
	(0.112)	(0.105)	(0.098)	(0.056)	(0.055)	(0.055)
Province FE	Yes	Yes	Yes	Yes	Yes	Yes
Year FE	Yes	Yes	Yes	Yes	Yes	Yes
Obs.	816	816	816	816	816	816
R²	0.572	0.558	0.546	0.559	0.551	0.545

***, **, and * indicate significance at the 1%, 5%, and 10% levels, respectively; standard errors are reported in parentheses.

Columns (4)-(6) include the mediator variables and the core independent variable simultaneously in the regression model. The coefficients of *Tech* (0.028), *Human* (0.024), and *Logis* (0.022) are all significantly positive at the 1% level, while the coefficient of *AD* remains significantly positive but decreases compared to the benchmark regression. These results indicate that agricultural technological progress, human capital, and logistics efficiency play partial mediation roles in the impact of agricultural digitalization on AEQ. Agricultural digitalization not only directly promotes the improvement of export quality but also indirectly enhances it through these three channels. The above results validate Hypothesis ***H***_**2**_.

### 4.5 Moderating role test

In reality, the agricultural digitalization plays a crucial role in enhancing AEQ. However, its effectiveness varies significantly across provinces, which is not only related to the development status of each province but also closely linked to the strength of local policy support and the degree of market openness. Based on this, this study analyzes the moderating role of policy support intensity and market openness in the process through which the agricultural digitalization affects AEQ. Table 8 introduces interaction terms between *AD* and moderator variables (*PolicySupport* and *MarketOpenness*). The coefficients of the interaction terms are 0.019 and 0.022, both significant at the 1% level, indicating that policy support and market openness can strengthen the promotion effect of agricultural digitalization on AEQ. The above results validate Hypothesis *H*_3_.

**Table 8:** Moderating role test results.

Variables	(1)*AEQ*	(1)*AEQ*
*AD*	0.038***	0.036***
	(0.006)	(0.006)
*AD×PolicySupport*	0.019***	
	(0.004)	
*AD×MarketOpenness*		0.022***
		(0.004)
*PolicySupport*	0.025***	
	(0.005)	
*MarketOpenness*		0.028***
		(0.005)
*GDP*	0.024***	0.023***
	(0.007)	(0.007)
*Scale*	0.012**	0.011**
	(0.006)	(0.006)
*RCA*	0.026**	0.025***
	(0.006)	(0.006)
*Industry*	−0.099***	−0.097***
	(0.023)	(0.023)
*FDI*	0.015***	0.014***
	(0.003)	(0.003)
*Climate*	0.010**	0.010**
	(0.003)	(0.003)
*LandScale*	0.015***	0.014***
	(0.003)	(0.003)
*FinSupport*	0.008**	0.007**
	(0.002)	(0.002)
*TradeEnv*	0.012***	0.011***
	(0.003)	(0.003)
*_cons*	8.012***	8.035***
	(0.062)	(0.063)
Province FE	Yes	Yes
Year FE	Yes	Yes
Obs.	816	816
R²	0.549	0.541

***, **, and * indicate significance at the 1%, 5%, and 10% levels, respectively; standard errors are reported in parentheses.

### 4.6 Heterogeneity analysis

Table 9 explores the heterogeneous impacts of agricultural digitalization on AEQ from three dimensions: regional distribution, agricultural attribute, and institutional environment. From the regional perspective (Columns 1–2), the coefficient of *AD* in Eastern China (0.059) is significantly higher than that in Midwest China (0.032), and both are statistically significant. This suggests that the promotion effect of agricultural digitalization is more pronounced in Eastern China, which benefits from better digital infrastructure and higher technological absorption capacity.

**Table 9:** Heterogeneity regression results.

Variables	(1) Eastern China	(2) Midwest China	(3)Grain Production Area	(4) Non-grain Area	(5) Marketization	(6) Low Marketization
*AD*	0.059***	0.032*	0.049**	0.035**	0.056***	0.029
	(0.007)	(0.006)	(0.006)	(0.007)	(0.006)	(0.006)
*GDP*	0.034***	0.021**	0.029***	0.022**	0.031***	0.018**
	(0.009)	(0.008)	(0.008)	(0.008)	(0.008)	(0.007)
*Scale*	0.020**	0.011*	0.017**	0.01	0.019**	0.009
	(0.007)	(0.006)	(0.007)	(0.006)	(0.007)	(0.006)
*RCA*	0.036***	0.020**	0.033***	0.021**	0.034***	0.019**
	(0.007)	(0.006)	(0.007)	(0.006)	(0.006)	(0.006)
*Industry*	−0.135***	−0.096***	−0.121**	−0.092***	−0.128***	−0.087***
	(0.029)	(0.023)	(0.026)	(0.024)	(0.027)	(0.022)
*FDI*	0.022***	0.012**	0.019***	0.013**	0.020***	0.011**
	(0.004)	(0.003)	(0.004)	(0.003)	(0.003)	(0.003)
*Climate*	0.015***	0.007**	0.014***	0.008**	0.013***	0.006**
	(0.004)	(0.003)	(0.003)	(0.003)	(0.003)	(0.003)
*LandScale*	0.022***	0.010**	0.020***	0.011**	0.021**	0.009**
	(0.004)	(0.003)	(0.004)	(0.003)	(0.003)	(0.003)
*FinSupport*	0.012***	0.005*	0.011***	0.006**	0.012***	0.005*
	(0.003)	(0.002)	(0.003)	(0.002)	(0.003)	(0.002)
*TradeEnv*	0.018***	0.009**	0.016***	0.010**	0.017***	0.008**
	(0.004)	(0.003)	(0.004)	(0.003)	(0.003)	(0.003)
*_cons*	7.826***	8.059***	7.863***	8.015***	7.795***	8.102***
	(0.067)	(0.058)	(0.063)	(0.059)	(0.061)	(0.057)
LR test	15.73		11.48		13.86	
Province FE	Yes	Yes	Yes	Yes	Yes	Yes
Year FE	Yes	Yes	Yes	Yes	Yes	Yes
Obs.	336	480	448	368	384	432
R²	0.586	0.473	0.551	0.468	0.572	0.459

***, **, and * indicate significance at the 1%, 5%, and 10% levels, respectively; standard errors are reported in parentheses.

In terms of agricultural attributes (Columns 3–4), the coefficient of *AD* in grain production areas (0.049) is larger than that in non-grain areas (0.035), indicating that agricultural digitalization has a stronger driving effect on AEQ in grain production areas with higher standardization levels. From the institutional environment perspective (Columns 5–6), high marketization level regions exhibit a larger coefficient (0.056) compared to low marketization level regions (0.029), implying that a more developed market system can better leverage the role of agricultural digitalization. The significant likelihood ratio (LR) test results further confirm the existence of significant heterogeneous effects across subgroups.

### 4.7 Extended analysis

Table 10 reports the quantile regression results at five quantiles (0.10, 0.25, 0.50, 0.75, and 0.90) to examine the heterogeneous effects of agricultural digitalization on agricultural export quality (AEQ) across different quality levels. The coefficient of AD exhibits a clear inverted U-shaped pattern: it increases from 0.023 at the 0.10 quantile to 0.068 at the 0.50 quantile (the peak value), and then declines to 0.037 at the 0.90 quantile. All coefficients are statistically significant.

**Table 10:** Extended analysis results.

Variables	(1) Q0.10 (Lower)	(2) Q0.25 (Lower-middle)	(3) Q0.50 (Middle)	(4) Q0.75 (Upper-middle)	(5) Q0.90 (Higher)
*AD*	0.023**	0.041***	0.068***	0.059***	0.037**
	(0.009)	(0.007)	(0.008)	(0.009)	(0.010)
*GDP*	0.015*	0.024**	0.036**	0.031***	0.022**
	(0.008)	(0.008)	(0.009)	(0.009)	(0.010)
*Scale*	0.007	0.013*	0.021**	0.018**	0.009
	(0.007)	(0.007)	(0.008)	(0.009)	(0.009)
*RCA*	0.018*	0.027***	0.043***	0.038**	0.025**
	(0.009)	(0.007)	(0.008)	(0.009)	(0.010)
*Industry*	−0.061**	−0.093***	−0.145**	−0.132***	−0.089***
	(0.024)	(0.025)	(0.028)	(0.030)	(0.032)
*FDI*	0.009*	0.015**	0.024***	0.021***	0.012**
	(0.004)	(0.004)	(0.005)	(0.005)	(0.005)
*Climate*	0.006	0.009*	0.017***	0.015***	0.008*
	(0.003)	(0.004)	(0.004)	(0.004)	(0.004)
*LandScale*	0.008*	0.015**	0.026***	0.023***	0.011**
	(0.004)	(0.004)	(0.005)	(0.005)	(0.005)
*FinSupport*	0.004	0.007*	0.013***	0.011***	0.006
	(0.002)	(0.003)	(0.003)	(0.003)	(0.003)
*TradeEnv*	0.007*	0.012**	0.020**	0.017***	0.009*
	(0.004)	(0.004)	(0.004)	(0.005)	(0.005)
*_cons*	8.087***	8.012***	7.853***	7.896***	8.035***
	(0.060)	(0.057)	(0.062)	(0.065)	(0.068)
Province FE	Yes	Yes	Yes	Yes	Yes
Year FE	Yes	Yes	Yes	Yes	Yes
Pseudo R2	0.295	0.382	0.567	0.534	0.369
Obs.	816	816	816	816	816

***, **, and * indicate significance at the 1%, 5%, and 10% levels, respectively; standard errors are reported in parentheses.

These results suggest that the promoting effect of agricultural digitalization is strongest for provinces with medium levels of AEQ. In provinces with relatively low export quality, limited digital infrastructure and weaker technological absorption capacity constrain the effectiveness of agricultural digitalization. In contrast, for provinces with higher export quality, the marginal impact of digitalization diminishes as these regions approach the quality frontier and require more advanced and specialized digital technologies to achieve further improvements. The pseudo *R*^*2*^ follows a similar pattern across quantiles, further supporting the robustness of the inverted U-shaped relationship.

## 5. Conclusions and implications

### 5.1 Main conclusions

This study empirically examines the impact of agricultural digitalization on agricultural export quality (AEQ) using panel data from 34 provincial administrative regions in China over the period 2001–2024. A comprehensive empirical framework is employed, including baseline regressions, mediation effect tests, moderating effect tests, heterogeneity analysis, endogeneity treatments, robustness checks, and quantile regression analysis. The main findings can be summarized as follows.

First, agricultural digitalization exerts a significantly positive and robust effect on improving AEQ. The core coefficient remains stable across multiple model specifications, ranging from 0.045 to 0.057 and remaining statistically significant at the 1% level. This result holds after introducing control variables and provincial and year fixed effects, as well as after addressing endogeneity using the two-stage least squares (2SLS) method, the Heckman two-step procedure, and the Bartik instrumental variable approach.

Second, the promotion effect operates through three partial mediation channels: agricultural technological progress, agricultural human capital improvement, and enhanced agricultural logistics efficiency. The mediation coefficients are 0.028, 0.024, and 0.022, respectively, and all are statistically significant at the 1% level.

Third, the impact of agricultural digitalization demonstrates significant heterogeneity across regions and development conditions. The effect is more pronounced in eastern China, major grain-producing regions, and areas with higher levels of marketization than in central and western regions, non–grain-producing areas, and regions with lower levels of marketization. This finding highlights the importance of digital infrastructure and institutional environments in enabling the benefits of agricultural digitalization.

Fourth, policy support and market openness play significant moderating roles in the relationship between agricultural digitalization and AEQ. Both factors strengthen the positive effect of agricultural digitalization, with interaction coefficients of 0.019 and 0.022, respectively, both significant at the 1% level.

Finally, the quantile regression results reveal an inverted U-shaped relationship between agricultural digitalization and AEQ. The promotion effect is strongest in provinces with medium export quality, with a coefficient of 0.068 at the 0.50 quantile. In contrast, the effect is weaker in provinces with lower export quality (0.023 at the 0.10 quantile) and those with higher export quality (0.037 at the 0.90 quantile). This pattern reflects differences in digital infrastructure endowments, technological absorption capacity, and the potential for quality upgrading across regions.

Overall, these findings demonstrate that agricultural digitalization serves as a key driver of AEQ upgrading in China. However, its effectiveness depends on multiple factors, including transmission mechanisms, regional characteristics, institutional environments, and the initial level of export quality.

### 5.2 Policy suggestions

Based on the above findings, several policy recommendations are proposed to better leverage agricultural digitalization in promoting improvements in AEQ.

First, efforts should be made to strengthen the foundational conditions for agricultural digital development. Governments should expand the coverage of high-quality digital infrastructure in agricultural production areas, particularly in regions with relatively weak development foundations. Priority should be given to improving network connectivity, data platforms, and other essential digital facilities. At the same time, it is necessary to further improve institutional systems related to agricultural product quality standards, certification, and traceability. Establishing a sound regulatory and institutional framework will provide clear market norms for the application of digital technologies and create a favorable environment for improving the quality and competitiveness of agricultural exports.

Second, policies should promote the deep integration of digital technologies across the entire agricultural value chain. Digital tools such as big data and the Internet of Things should be widely applied to optimize key processes including production, breeding, processing, storage, and transportation. Such integration can facilitate precision management and data-driven decision-making throughout the agricultural production system. In addition, greater emphasis should be placed on strengthening human capital support. Through large-scale training programs for professional farmers and the introduction of specialized digital talent, policymakers can enhance the capacity of agricultural producers to adopt and utilize digital technologies effectively. Meanwhile, accelerating the development of smart agricultural logistics systems is also essential. Investments in cold chain infrastructure, storage facilities, and digital logistics platforms can improve distribution efficiency, reduce post-harvest losses, and ensure the quality and freshness of agricultural products in international markets.

Third, policy design should reflect regional heterogeneity in the development of agricultural digitalization. Regions with well-developed digital infrastructure and mature market mechanisms should be encouraged to explore deeper digital integration across the entire agricultural industry chain and to pursue business model innovation aimed at higher value-added agricultural exports. In contrast, regions with relatively weaker foundations should prioritize the adoption of practical, low-cost, and easy-to-implement digital solutions. Additional policy support, such as improved market access and financial services, can help stimulate endogenous development incentives in these regions. Such differentiated policy approaches can enable each region to pursue the most suitable digital transformation path based on its specific resource endowments and development conditions.

Finally, a long-term institutional mechanism should be established to support the sustained development of agricultural digitalization. This includes strengthening interdepartmental coordination, improving data-sharing systems, and enhancing policy synergy across relevant sectors. Governments should also leverage the guiding role of public investment to attract greater participation from private capital and encourage innovation in agricultural digital technologies. At the same time, a dynamic monitoring and evaluation system should be established to assess policy implementation and track progress in digital transformation. This will allow policymakers to adjust strategies in a timely manner and ensure the continuous improvement of China’s agricultural export quality and international competitiveness.
